# Supplementary material for: Content-rich biological network constructed by mining PubMed abstracts
Source: BMC Bioinformatics. 2004 Oct 8;5:147. doi: 10.1186/1471-2105-5-147 (PMC528731; doi:10.1186/1471-2105-5-147)
Supplement: Additional File 2 — The original results of the above study (non-essential files are deleted to keep the file size under the limit set by BMC bioinformatics). [file 1471-2105-5-147-S2.bz2 › chilibotAdditionalFile2/dip05/490ID10428808E10653/html/AR_TMF1.html]

 


 **AR** and **TMF1** 
  
Found 4 abstracts in PubMed, retrieved 4.  
 

 What does Google say? 
 PDF only 
| .edu only 

---

**Interactive relationship** (e.g. stimulation, inhibition, etc)

**Stimulatory relationship**- ARA160, an  **AR**  N terminal interacting protein also known as TATA element modulatory factor  [ **TMF1** ]  TMF  [ **TMF1** ] , was subsequently shown to cooperate with ARA70 in enhancing  **AR**  activity.  Ref: 11501969 J Biol Regul Homeost Agents< MedlineTA>J Biol Regul Homeost Agents,
**Neutral relationship**- The far Western blotting and co immunoprecipitation assays demonstrate that the  **AR**  can interact directly with ARA160 TMF  [ **TMF1** ] .  Ref: 10428808 J Biol Chem, 1999
- Here we show that hbrm hSNF2 alpha and BRG 1 hSNF2 beta, the ATPase subunits of the human SNF SWI complexes, specifically associate in vitro and in vivo with TATA element modulatory factor  [ **TMF1** ]  TMF  [ **TMF1** ]  ARA160, which has been described as a binding protein to and coactivator for the androgen receptor  [ **AR** ] .  Ref: 12044884 FEBS Lett, 2002

**Non-interactive relationship** (e.g. studied together, co-existance, homology, etc.)

- Our results revealed nearly constant expression of  **AR**  and heterogeneous expression of  **AR**  co factors, with increased expression of PIAS1 and Ran ARA24, decreased expression of ELE1 ARA70, and no change in  **TMF1**  ARA160, ARA54, SRC1, or TRAP220.  Ref: 12368219 Am J Pathol, 2002
